# Theory of transformation-mediated twinning

**DOI:** 10.1093/pnasnexus/pgac282

**Published:** 2022-12-07

**Authors:** Song Lu, Xun Sun, Yanzhong Tian, Xianghai An, Wei Li, Yujie Chen, Hualei Zhang, Levente Vitos

**Affiliations:** Applied Materials Physics, Department of Materials Science and Engineering, Royal Institute of Technology, Brinellvägen 23, Stockholm, SE-10044, Sweden; Applied Materials Physics, Department of Materials Science and Engineering, Royal Institute of Technology, Brinellvägen 23, Stockholm, SE-10044, Sweden; State Key Laboratory for Mechanical Behavior of Materials, Frontier Institute of Science and Technology, Xi’an Jiaotong University, Xi’an 710049, China; Key Laboratory for Anisotropy and Texture of Materials (Ministry of Education), School of Materials Science and Engineering, Northeastern University, Shenyang 10819, China; School of Aerospace, Mechanical & Mechatronic Engineering, The University of Sydney, Camperdown Sydney, NSW 2006, Australia; Applied Materials Physics, Department of Materials Science and Engineering, Royal Institute of Technology, Brinellvägen 23, Stockholm, SE-10044, Sweden; School of Aerospace, Mechanical & Mechatronic Engineering, The University of Sydney, Camperdown Sydney, NSW 2006, Australia; School of Mechanical Engineering, University of Adelaide, Adelaide, SA 5005, Australia; State Key Laboratory for Mechanical Behavior of Materials, Frontier Institute of Science and Technology, Xi’an Jiaotong University, Xi’an 710049, China; Applied Materials Physics, Department of Materials Science and Engineering, Royal Institute of Technology, Brinellvägen 23, Stockholm, SE-10044, Sweden; Department of Physics and Astronomy, Division of Materials Theory, Uppsala University, Uppsala, Box 516, SE-75121, Sweden; Research Institute for Solid State Physics and Optics, Wigner Research Center for Physics, Budapest H-1525, Hungary

**Keywords:** twinning, martensitic transformation, stacking fault, metastable alloy

## Abstract

High-density and nanosized deformation twins in face-centered cubic (fcc) materials can effectively improve the combination of strength and ductility. However, the microscopic dislocation mechanisms enabling a high twinnability remain elusive. Twinning usually occurs via continuous nucleation and gliding of twinning partial dislocations on consecutive close-packed atomic planes. Here we unveil a completely different twinning mechanism being active in metastable fcc materials. The transformation-mediated twinning (TMT) is featured by a preceding displacive transformation from the fcc phase to the hexagonal close-packed (hcp) one, followed by a second-step transformation from the hcp phase to the fcc twin. The nucleation of the intermediate hcp phase is driven by the thermodynamic instability and the negative stacking fault energy of the metastable fcc phase. The intermediate hcp structure is characterized by the easy slips of Shockley partial dislocations on the basal planes, which leads to both fcc and fcc twin platelets during deformation, creating more twin boundaries and further enhancing the prosperity of twins. The disclosed fundamental understanding of the complex dislocation mechanism of deformation twinning in metastable alloys paves the road to design novel materials with outstanding mechanical properties.

Significance Statement The present work reveals a fundamental dislocation mechanism of deformation twinning in metastable fcc materials. We put forward the novel TMT mechanism, which explains the unusually high twinnability and the outstanding mechanical properties of these materials. Our findings highlight the critical role played by the negative stacking fault energy in dictating dislocation behaviors, which unfortunately is not accessible by current experimental methods. The present work advances the knowledge on the theory of plasticity and guides future alloy design towards desired mechanical performance.

## Introduction

Plastic properties of metallic materials are mainly mediated by the nucleation and motion of dislocations. When plastic deformation is solely accommodated by dislocation slips, the strength-ductility dilemma is generally inevitable since the conventional metallurgical strengthening methods via generating internal barriers for dislocation motion cause a sacrifice in ductility (1, 2). Introducing additional deformation mechanisms like strain stimulated phase transformation or twinning is an effective strategy for attaining both high strength and excellent ductility as successfully demonstrated in the so-called transformation-induced plasticity (TRIP) and twinning-induced plasticity (TWIP) alloys (3–5). In the classical theory of plasticity for face-centered cubic (fcc, γ) metals, deformation twinning (DT) is realized by layer-by-layer nucleation and gliding of Shockley partial dislocations on consecutive close-packed planes, which gradually reverses the stacking sequence of the close-packed planes from fcc (...ABCABC...) to twin (...CBACBA...). More specifically, the stacking order changes with increasing partial slips as in the following,...ABCABCABC... (fcc) →...ABCA|CABCA... (stacking fault (SF), “|” the SF plane) →...ABC$\bigcirc\hskip-6pt {A}$|C|$\bigcirc\hskip-6pt {B}$CAB... (three-layer twin nucleus with twin boundary mirror planes circled) →...ABC$\bigcirc\hskip-6pt {A}$|C|B|$\bigcirc\hskip-6pt {A}$BC... (twin thickening); while deformation-induced martensite transformation (DIMT) from fcc to hexagonal close-packed (hcp, ε) structure (γ → ε) by regular partial movements on every other close-packed planes, i.e.,...ABCABCABC... (fcc) →...ABCA|CABCA... (SF)→...ABCA|CA|CAB... (four-layer ε nucleus) →...ABCA|CA|CA|C... (ε thickening). Since the partial dislocations for DT and DIMT are identical, in the extant theories of plasticity as well in practice (5, 6), the above two mechanisms are considered exclusive to each other, operating in materials with different ranges of stacking fault energies (SFEs) or local SFEs due to chemical variations (7–9). Empirically, a critical measured SFE value [∼10 to 20 mJ m^−2^, depending on chemistry (5)] is often placed for the deformation mode transition from DIMT to DT. However, in many metallic systems (3,4,7,10–20), DIMT and DT have been simultaneously observed in the same grains, forming a unique alternate lamellar structure composed of nano-thicknessed fcc twin (γ_tw_) and hcp laths. Such nanoscaled deformation structure is crucial, because both the γ/γ_tw_ twin and the γ/ε phase boundaries can not only carry significant plasticity, but also interact strongly with dislocations, catalyzing the remarkably enhanced work-hardening capacity (1). However, the concurrence of DIMT and DT challenges the current knowledge about the underlying microscopic mechanism, which remains elusive even after about half a century since its early observation (12). The highly unsatisfying understanding of the deformation dynamics in these metals and alloys has limited the ability to provide an accurate prediction of the composition-structure-property relationships, and restricted the capability of designing materials with superior mechanical properties.

Here we zoom in the transition zone between DT and γ → ε DIMT to shed light on the atomistic processes responsible for the deformation mode change. We identify an unorthodox twinning mechanism in thermodynamically unstable (metastable) fcc metals and alloys, which does not follow the classical γ → γ_tw_ layer-by-layer twinning (cTW) path. Using quantum mechanical density functional theory (DFT) calculations, we show that in such systems DT can be realized through an intermediate γ → ε DIMT by sequentially activated groups of partial dislocations, which lead to a two-step γ → ε → γ_tw_ twinning process. This novel transformation-mediated twinning (TMT) mechanism originates from the metastability of the fcc lattice and from the basal plane shear instability of the hcp lattice. Remarkably, the TMT mechanism can provide a profound understanding for the pronounced twinning activities in metastable alloys. We pinpoint to the critical factors controlling the deformation mode transition from DIMT to DT, which enable alloy design targeting exceptional mechanical properties to maximally harvest the TWIP/TRIP effect.

## Results

### Classical twinning versus γ → ε martensitic transformation

**Fig. 1.**
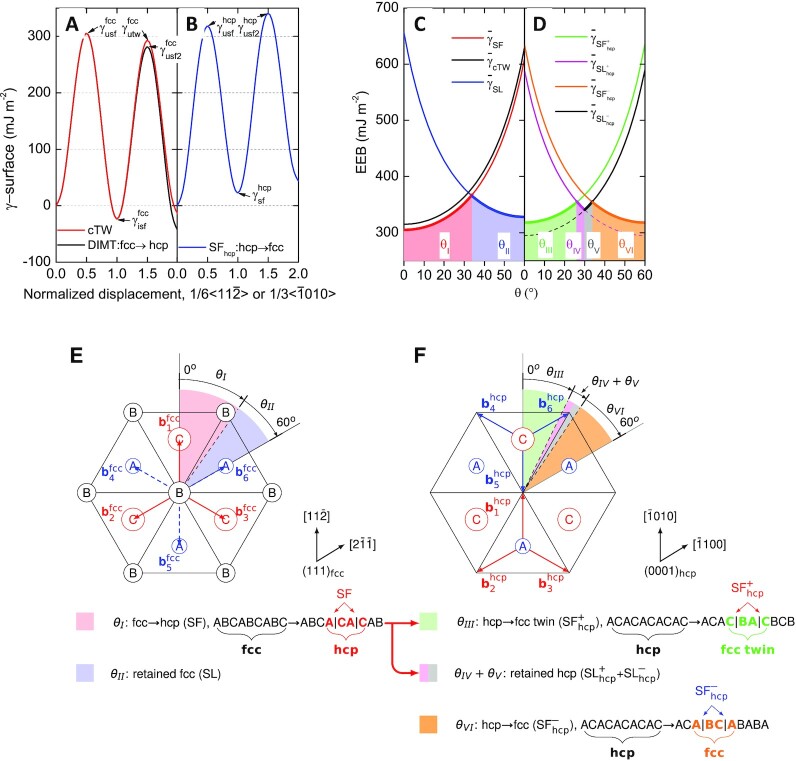
γ-surfaces and orientation dependent energy barriers for deformation mechanisms in fcc and hcp CrCoNi. (A and B) The room temperature γ-surfaces for γ → ε DIMT and cTW in fcc and for the basal stacking fault (SF_hcp_) formation in hcp lattices, respectively. (C) The orientation dependent EEBs for SF formation, cTW, and SL in fcc lattice. (D) The orientation dependent EEBs for stacking faults (SF$^+_{\rm hcp}$ and SF$^-_{\rm hcp}$) and dislocation slips (SL $^+_{\rm hcp}$ and SL$^-_{\rm hcp}$) on the basal planes in hcp lattice. The lowest EEBs corresponding to the favored deformation modes are highlighted by thick lines. (E and F) The orientation dependent deformation modes are illustrated on the (111)_fcc_ planes in fcc and the (0001)_hcp_ basal planes in hcp lattices, respectively. Details about figure preparation are available in Note S4.

We take the extensively studied metastable CrCoNi medium-entropy alloy (MEA) to investigate the competition between γ → ε DIMT and DT, but emphasize that the discussion presented here applies to most of the metastable metals and alloys. This alloy possesses a number of intriguing mechanical properties due to its remarkable twinnability, especially at cryogenic conditions (21, 22). The generalized stacking fault energies (GSFEs, γ-surfaces) at room temperature for the fcc and hcp phases of CrCoNi are shown Figs. 1(A and B), respectively. The γ-surfaces for the fcc phase (Fig. 1A) show the energy barrier (the unstable stacking fault energy, $\gamma ^{\rm fcc}_{\rm usf}$) to create an intrinsic SF with formation energy $\gamma ^{\rm fcc}_{\rm isf}$ by a leading partial, and the energy barrier (the unstable twin fault energy, $\gamma ^{\rm fcc}_{\rm utw}$) for the three-layer twin nucleus. The energy barrier for γ → ε DIMT is given by $\gamma ^{\rm fcc}_{\rm usf2}$, corresponding to the barrier for generating the second SF two atomic layers away from the existing one. The γ-surface for the hcp phase (Fig. 1B) describes the energy barrier ($\gamma ^{\rm hcp}_{\rm usf}$) for creating a basal plane SF with formation energy $\gamma ^{\rm hcp}_{\rm sf}$. The calculated fcc SFE is negative at room temperature ($\gamma ^{\rm fcc}_{\rm isf}= -21$ mJ m^−2^), which signals that the hcp structure is energetically favored from the view that a SF is structurally equivalent to a two-layer hcp phase. This is in line with previous theoretical results (7, 23, 24), as well as the experimental observations showing a large amount of SFs already at early deformations (7, 18, 19, 22). Furthermore, starting from an existing SF, the hcp nucleation and growth have a lower energy barrier to overcome than that for the classical layer-by-layer twinning nucleation ($\gamma _{\rm usf2}^{\rm fcc}\lt \gamma _{\rm utw}^{\rm fcc}$, Fig. 1A). Theoretically, the competition among deformation modes in a fcc material can be quantitatively measured by the effective energy barriers (EEBs) based on the γ-surface (6,24). The EEBs for SF formation (the same as for γ → ε DIMT), cTW, and full dislocation slip (SL) were defined as

(1) $$\begin{eqnarray*}
\overline{\gamma }_{\rm SF}(\theta )=\gamma ^{\rm fcc}_{\rm usf}/\cos {\theta },
\end{eqnarray*}
$$

(2) $$\begin{eqnarray*}
\overline{\gamma }_{\rm cTW}(\theta )=(\gamma ^{\rm fcc}_{\rm utw}-\gamma ^{\rm fcc}_{\rm isf})/\cos {\theta },
\end{eqnarray*}
$$

(3) $$\begin{eqnarray*}
\text{and } \overline{\gamma }_{\rm SL}(\theta )=(\gamma ^{\rm fcc}_{\rm usf}-\gamma ^{\rm fcc}_{\rm isf})/\cos {(60^{\circ }-\theta )},
\end{eqnarray*}
$$

respectively. Here, θ measures the angle between the resolved shear stress on the (111)_fcc_ slip planes and the Burgers vector for leading (twinning) partials to account for the unidirectional nature of DT/DIMT in fcc structure (25, 26). In the present work, θ is referred to *a*/6(11$\bar{2}$) on (111)_fcc_ plane and spans 0° to 60° according to the symmetry of the (111)_fcc_ planes (Fig. 1E). The preferred deformation mode is decided by the lowest EEB (6). We must emphasize here that the above EEB for twinning ($\overline{\gamma }_{\rm cTW}(\theta )$) is defined according to the classical layer-by-layer twinning route (6, 24), therefore, $\overline{\gamma }_{\rm cTW}(\theta )\gt \overline{\gamma }_{\rm SF}(\theta )$ implies that energetically leading partial dislocations will avoid nucleation and gliding on the nearest neighboring slipping planes, instead, they prefer to slip on distant planes and create separated SFs. This is the case of fcc CrCoNi (Fig. 1C). Crystal orientation can change the preference of SF and SL through altering the resolved shear stresses on specific slip systems according to the Schmid law. From Fig. 1C, we see that SF and ε formation occur for small θ (θ_I_= 0° to 34°) i.e., when the direction of the resolved shear stress is close to ${\bf b}^{\rm fcc}_1$; while SL is the favored deformation mode at larger θ (θ_II_ = 34° to 60°) when the resolved shear stress is close to the direction of ${\bf b}^{\rm fcc}_6$ and the trailing partial is activated to follow the leading one. However, the competition between cTW and γ → ε DIMT is not affected by θ because they are accomplished through the same type of partial dislocations. Overall, the above prediction that DIMT is preferred over twinning in terms of EEBs is apparently at odds with the indisputable observation of a large amount of deformation twins. Extant theories of twinning nucleation based on the cTW path, when modified by considering local chemical variations (7–9), a substantial affine shear (24), or local adiabatic heating (4), may turn twinning favorable locally in multicomponent solid solutions, but all these scenarios fail to explain the observed low fraction of ε martensite, and the characteristic arrangement of γ/γ_tw_/ε lamellar structure (7, 18–20, 22), nor they can be universally applied to explain similar observations in other metals with negative SFE like pure fcc Co ($\gamma ^{\rm fcc}_{\rm isf}=-97$ mJ m^−2^, Table S1) (10).

### Phase transformation-mediated twinning

We observe that γ → ε DIMT itself is not an effective avenue for strain accommodation, considering that only partials on every other slip planes are involved in the process. Therefore, the fresh deformation-induced ε lamella formed in the fcc matrix is also subject to strains during further deformation, which is indeed evidenced by the pronounced dislocation activities observed in the ε martensite, particularly at large strains (3). However, even before the ε phase gets thickened and expanded, dislocation activities on the basal planes are expected. Due to the strictly complied Shoji-Nishiyama (S-N) orientation relationship (OR) (19), i.e., (111)_fcc_//(0001)_hcp_ and $\lt 11\bar{2}$>_fcc_//<$\bar{1}010\gt $_hcp_, the dominant dislocation activities occur on the hcp basal planes (27). However, due to the...ACAC... stacking, fundamental difference arises for the activation of the leading partials in the hcp lattice compared to that in the fcc one, regarding the crystal orientation dependence. In the fcc structure (stacking sequence...ABCABC...), on all three consecutive (111)_fcc_ planes there is only one set of 1/6$\lt 11\bar{2}$> Burgers vectors (${\bf b}_{1}^{\rm fcc}$, ${\bf b}_{2}^{\rm fcc}$, and ${\bf b}_{3}^{\rm fcc}$, deviated by 120°, Fig. 1E) for the leading partials, which can create SFs. The rest three Burgers vectors (${\bf b}_{4}^{\rm fcc}$, ${\bf b}_{5}^{\rm fcc}$, and ${\bf b}_{6}^{\rm fcc}$) are for trailing partials, which recover SF to fcc. In contrast, in the hcp lattice there are two sets of Burgers vectors for the leading partials gliding on the basal planes C (${\bf b}_{1}^{\rm hcp}$, ${\bf b}_{2}^{\rm hcp}$, and ${\bf b}_{3}^{\rm hcp}$) and A (${\bf b}_{4}^{\rm hcp}$, ${\bf b}_{5}^{\rm hcp}$, and ${\bf b}_{6}^{\rm hcp}$), respectively, which deviate from each other by 60° (Fig. 1F). In other words, there are six equivalent glide directions on the hcp basal planes to generate SFs (denoted as SF_hcp_). Explicitly, in the fcc lattice, the atomic movement carried out by the leading partials is always A→ B→ C→ A; whereas the reverse slip sequence A→ C→ B→ A along the trailing/anti-twinning directions (i.e., from...ABCABC... to...ABC|CAB...) is prohibited by the significantly higher slip barrier. On the other hand, in the hcp lattice, the A→ B→ C→ A (via slips of partial dislocations with Burgers ${\bf b}_{1}^{\rm hcp}$, ${\bf b}_{2}^{\rm hcp}$, and ${\bf b}_{3}^{\rm hcp}$, Fig. 1F) and A→ C→B→ A (via slips of partial dislocations with Burgers ${\bf b}_{4}^{\rm hcp}$, ${\bf b}_{5}^{\rm hcp}$, and ${\bf b}_{6}^{\rm hcp}$, Fig. 1F) slip sequences are equivalent and can be realized on the C and A slip planes, respectively. We assign the A→ B→ C→ A and A→ C→B→ A slip sequences with the + and - signs, respectively, and the SFs formed by the + and - slip sequences are denoted as SF$^{+}_{\rm hcp}$ (i.e.,...ACACACAC...→...ACAC|BABA...) and SF$^{-}_{\rm hcp}$ (i.e.,...ACACACAC...→...ACACA|BCB...), respectively. In accordance with the hcp stacking sequence, under a specific resolved shear stress, the leading partial dislocations with the same Burgers vector are restricted to slip on every other (0001)_hcp_ planes, which actually leads to the ε → γ phase transformation. On each hcp basal plane, the SF_hcp_ formation competes with the full dislocation slip (denoted as SL_hcp_) and the corresponding EEBs for the ± slip sequences are

(4) $$\begin{eqnarray*}
\overline{\gamma }_{\rm SF^{+}_{\rm hcp}}(\theta )=\gamma ^{\rm hcp}_{\rm usf}/\cos {\theta },
\end{eqnarray*}
$$

(5) $$\begin{eqnarray*}
\overline{\gamma }_{\rm SL^{+}_{\rm hcp}}(\theta )=(\gamma ^{\rm hcp}_{\rm usf}-\gamma ^{\rm hcp}_{\rm sf})/\cos {(60^{\circ }-\theta }),
\end{eqnarray*}
$$

and,

(6) $$\begin{eqnarray*}
\overline{\gamma }_{\rm SF^{-}_{\rm hcp}}(\theta )=\gamma ^{\rm hcp}_{\rm usf}/\cos {(60^{\circ }-\theta }),
\end{eqnarray*}
$$

(7) $$\begin{eqnarray*}
\overline{\gamma }_{\rm SL^{-}_{\rm hcp}}(\theta )=(\gamma ^{\rm hcp}_{\rm usf}-\gamma ^{\rm hcp}_{\rm sf})/\cos {\theta }.
\end{eqnarray*}
$$

Here, $\gamma ^{\rm hcp}_{\rm sf}$ and $\gamma ^{\rm hcp}_{\rm usf}$ are the hcp stable and unstable SFEs, respectively (Fig. 1B). θ is measured from the slip direction leading to the positive slip sequence [($\bar{1}$010) in Fig. 1F], which is equivalent with the twinning direction in the fcc structure [(11$\bar{2}$) in Fig. 1E].

For the hcp phase of CrCoNi, the obtained EEBs (Fig. 1D) indicate that both SF$^{+}_{\rm hcp}$ and SF$^{-}_{\rm hcp}$ are the primary slip modes for low (θ_III_ = 0° to 26°) and high (θ_VI_ = 34° to 60°) angles, accomplished by partial dislocations with Burgers vector ${\bf b}_{1}^{\rm hcp}$ on C and ${\bf b}_{6}^{\rm hcp}$ on A planes, respectively (Fig. 1F). Full dislocations (SL$^{+}_{\rm hcp}$ and SL$^{-}_{\rm hcp}$) are restricted within very narrow orientation windows, namely θ_IV_ = 26° to 30° on C and θ_V_ = 30° to 34° on A when trailing partials are preferred and slip after the leading ones. Hence, the hcp phase of CrCoNi is strongly prone to SF_hcp_ formation, i.e., to the ε → γ phase transformation. Notably, this phase transformation is insensitive to crystal orientation, which is opposite to the unidirectional γ → ε DIMT and cTW processes in the fcc lattice (25, 26, 28). It is very important to recognize that the ε → γ transformations in the θ_III_ and θ_VI_ regions yield fcc products exactly in the twin relationship (Fig. 1F), because they are realized by the positive and negative slip sequences, respectively. Therefore, such geometrical features increase the likelihood of activation of leading partials with all six Burgers vectors in the hcp crystallines during deformation, especially under severe plastic deformations due to the complex shear stresses from multiple directions (29), which will generate nano thickness γ and γ_tw_ lamellar simultaneously, depending on the combination of the Burgers vectors of the partials. It is worth mentioning that the presently disclosed susceptibility of hcp lattice to SF$_{\rm hcp}^{+/-}$ formation should play important role in the observed phase reversion from the pressure-induced hcp phase to the metastable fcc phase during decompression of CrCoNi-based high entropy alloys (HEAs) (30, 31).

Based on the above results, we can draw a unified picture of the deformation processes for fcc CrCoNi. First, the pristine fcc grains with the primary slip plane having a small θ angle (in the range of θ_I_ = 0° to 34°) deform extensively by SFs, which will be manifested as the γ → ε DIMT when the maximum SF density [i.e., SFs on every other (111)_fcc_ planes,...ABCABCABCABCAB... (γ) →...ABC/A|CA|CA|CA|C/ABC... (γ/ε/γ)] is reached with increasing strains and the number of slips. Often, the ε lamellar are not perfect in stacking, and can be seen as multiple SFs. This step is driven by the energy decrease due to the negative $\gamma ^{\rm fcc}_{\rm isf}$. In other words, SFs avoid to form on the nearest-neighboring layers to maximize the energy decrease of the system, otherwise, SFs on the nearest-neighboring layers will result in two coherent twin boundaries, which together have the same energy density as a single stacking fault. Clearly, the later case is not thermodynamically prevailing when the SFE is negative. Then, the deformation-induced ε/SFs lamellar embedded in the fcc matrix further deform to release the local stress concentrations with increasing stains (3, 32). This is realized preferentially along the basal planes, which serve as the primary slip planes as the (111)_fcc_ planes because of the S-N OR. Additionally, it is well documented that the basal slips are remarkably easier than other slips involving dislocations with 〈$\bf c$〉 character in hcp metals, particularly when $\gamma _{\rm sf}^{\rm hcp}$ is small (33). Here, we measure the compatibility for activation of the leading partials of the same Burgers vector sequentially in the fcc matrix and in the hcp martensite by the relative EEB difference, $\delta _{\rm usf}^{\rm hcp-fcc}\equiv (\overline{\gamma }_{\rm SF_{hcp}^{+}}-\overline{\gamma }_{\rm SF})/\overline{\gamma }_{\rm SF}=(\gamma _{\rm usf}^{\rm hcp}-\gamma _{\rm usf}^{\rm fcc})/\gamma _{\rm usf}^{\rm fcc}$. For CrCoNi, $\delta _{\rm usf}^{\rm hcp-fcc}$ is as small as ∼6% (Table S1), which suggests that the critical resolved shear stress for the hcp basal SF formation can be easily reached at the same spot as that nucleates SF in the fcc lattice upon further loading. For θ in the range of 0° to 26° (θ_III_ in Fig. 1F), SF$^{+}_{\rm hcp}$s are formed by the positive slips; and consequently, they transform the fresh hcp lamella *forward* to the fcc twin, viz.,

1. ...ABC/ACACACAC/ABC... (γ/ε/γ) →
2. ...ABC|B/ABABABA/BCA... ($\gamma /\rm SF/\varepsilon /\gamma )\rightarrow$
3. ...AB$\bigcirc\hskip-6pt {C}$|BA|C/BCBCB/CAB... (γ/γ_tw_/ε/γ) →
4. ...AB$\bigcirc\hskip-6pt {C}$|BA|CB|A/CAC/ABC... (γ/γ_tw_/ε/γ) →
5. ...AB$\bigcirc\hskip-6pt {C}$|BA|CB|AC|B$\bigcirc\hskip-6pt {A}$BCA... (γ/γ_tw_/γ),

and thus, realizing the novel TMT mechanism. Depending on the positions of slip planes and the number of the slips, the combination of the lamellar varies. With further increasing strains, full dislocations in the fcc twins are activated to accommodate deformation ( Note S1). Immediately, the above processes explain why the strain-induced hcp phase in CrCoNi cannot grow in thickness, but remains limited to nanosize, even through it is thermodynamically stable (7, 18–20). Theoretically, if all the deformation-induced hcp lamellar with orientations in the θ_III_ region transfer to γ_tw_, only the hcp fractions developed in the fcc grains with θ in the range of θ_I_ to θ_III_ who deform by full dislocation slips on the basal planes can survive. If one further considers the strong texture development and grain rotation towards twinning favorable direction during deformation (18,19), very less hcp phase is expected to be reserved in actual observation. This perfectly explains why only a modest amount of hcp (∼3%) was observed at the fracture strain in CrCoNi MEA (19).

**Fig. 2.**
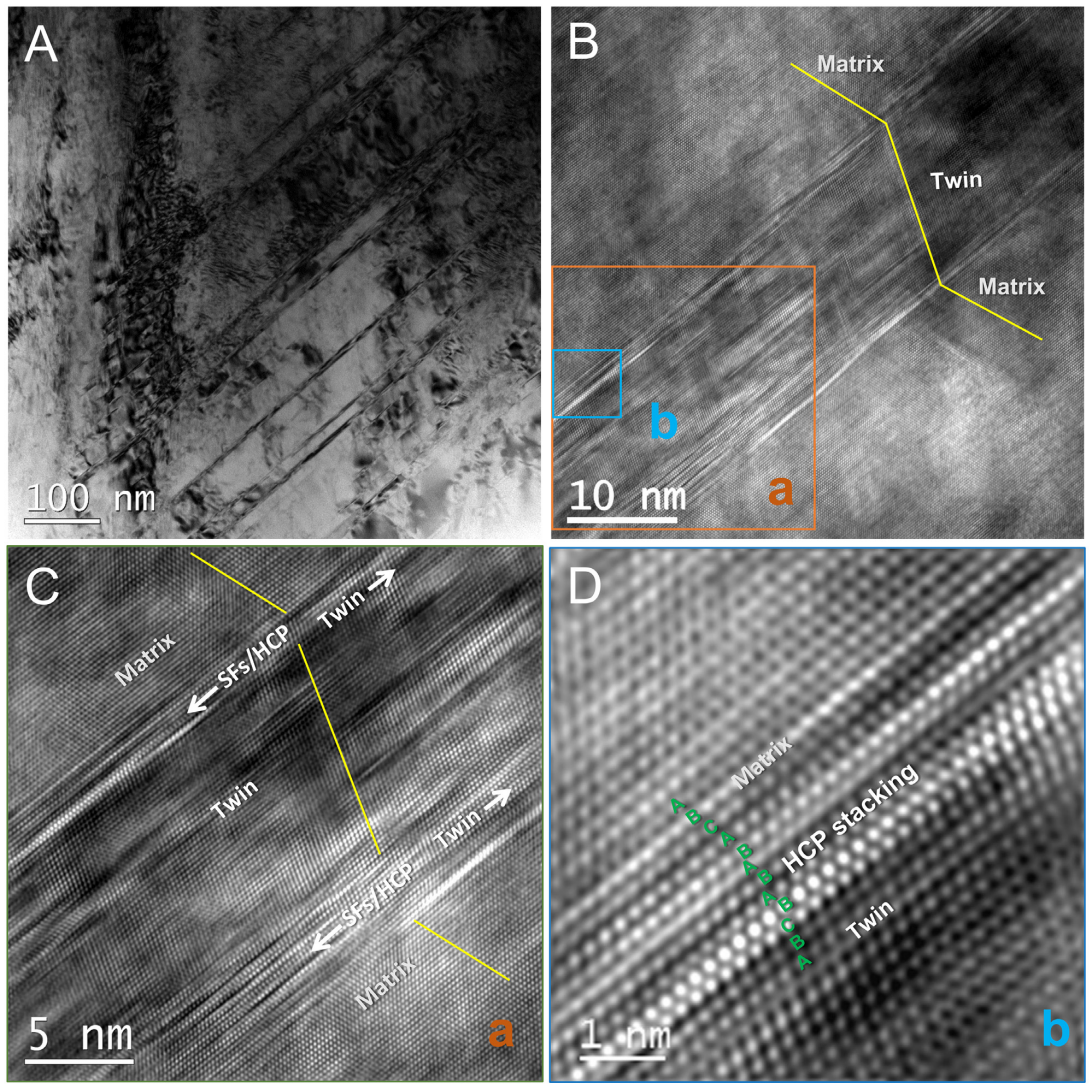
Typical deformation microstructures in a Co_32.3_Cr_36.3_Ni_31.4_ MEA after room temperature tensile testing till necking. (A) Bright field image and selected area diffraction. (B) The platelets are composed of lamellar structure of γ, γ_tw_, $\varepsilon (\rm SFs)$, and γ. (C) Enlarged figure of area a in (B). (D) High magnification image of area b in (B) showing the stacking sequence of...ABCA/BABA/B|CBA... ($\gamma /\varepsilon /\rm SF/\gamma _{\rm tw}$).

**Fig. 3.**
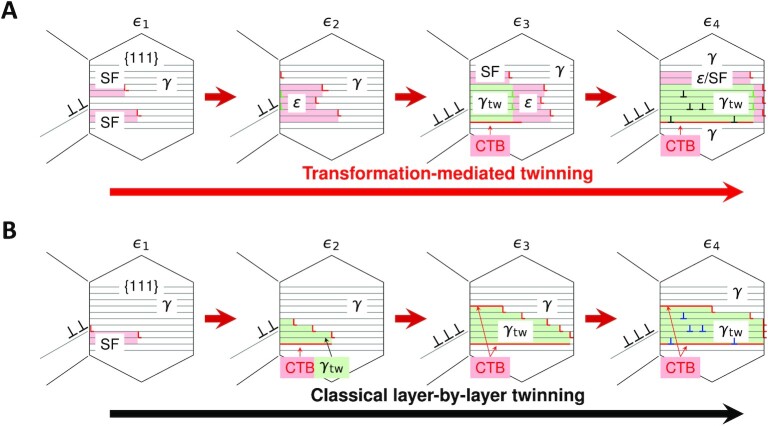
Schematics of deformation structure evolution with increasing strain levels (ϵ_1_ to ϵ_4_) via different twinning mechanisms with partials nucleated from grain boundaries. (A) Transformation-mediated twinning, showing the γ → SF/ε → γ_tw_ two-step twinning process, the formation of the characteristic γ/ε/γ and γ/γ_tw_/ε/γ deformation lamellar structures. (B) Classical layer-by-layer twinning in thermodynamically stable fcc materials with positive SFE.

The TMT mechanism provides an universal explanation for the observed γ/ε/γ and γ/γ_tw_/ε/γ lamellar structures in various metastable metals and alloys including the room-temperature fcc Co (10), Co-rich alloys (11), stainless steels (e.g., 304) (12, 13), high-Mn TWIP/TRIP steels (14–16), dual-phase fcc+hcp Cr_10_Mn_30_Fe_50_Co_10_ (3, 4) and single-phase CrCoNi-based high-entropy alloys (HEAs) (7, 17–20). We also demonstrate such typical microstructures in a Co_32.3_Cr_36.3_Ni_31.4_ MEA in Fig. 2. This alloy is designed to have a slightly more negative SFE than the equiatomic CrCoNi MEA to promote more DIMT (34). The fcc twins are often observed in immediate contact with the hcp/SFs plates (see Figure S3), similarly as in the above alloys (7, 18–20), which cannot be incontestably solved by existing theories based on the cTW route (7–9, 24), but can be well expected within the TMT picture because twins are thickened through consuming newly formed SFs/hcp platelet (Fig. 3A). Additionally, an obvious evidence for the TMT can be found in Figs. 2(B to D), which show that the fine hcp phase is in the immediate front of γ_tw_ in the same lamella of several atomic layers, as inferred from the present TMT mechanism (Fig. 3A). Similar experimental results were reported for CrCoNi MEA at room temperature and more hcp phase was observed at cryogenic temperature (18,19). Furthermore, the deformation structure evolution illustrated in Fig. 3(A) is also supported by molecular dynamics simulations in HEAs with negative SFEs (35, 36). Choi et al. (37) showed that separated SFs occur first, followed by micro-twins in simulation of CrMnFeCoNi nanowire under uniaxial tensile testing along the [110] direction. Areas with densely populated SFs, which can be seen as imperfect hcp phase were observed to transform to twin with increasing strain. Zheng et al. (35) reported a twinning pathway following the SF-hcp-twin steps in tensile simulation of a non-equiatomic CrMnFeCoNi HEA with negative SFE. The characterized lamellar structure of alternate SFs, ε, and γ_tw_ platelets were also observed in molecular dynamics simulations (35, 36).

When the SFE is positive, twin nucleation and thickening prefer the cTW path in order to avoid extra energy cost (Fig. 3B). This is also because the formation energy of coherent twin boundary (CTB) is about half of the SFE; consequently, increasing twin thickness according to the cTW mechanism does not require additional formation energy for the CTBs. In contrast, twinning according to the TMT mechanism is catalyzed by the energy gain (negative SFE) in the first step, which drives the extensive nucleation of SFs and fine ε phases, both homogeneously and heterogeneously; and then, the second step of TMT, the ε → γ_tw_ transformation, is assisted by the favorable basal slips in the ε martensite, i.e., other slip systems in the hcp structure are much more difficult. The above facts account for the exceptional twinnability in metastable fcc alloys, which provides the general rationale for the observed relationship between the SFE and twinning prosperity relationship. Additionally, since all six slip directions on the basal planes can transfer the ε martensite to γ or γ_tw_, this characteristic of the hcp structure renders that the complicated stress environment during deformation can create dense basal plane SFs, i.e., SF$^{-}_{\rm hcp}$ and SF$^{+}_{\rm hcp}$, which divide and transfer the intermediate hcp martensite into fine scale γ and γ_tw_ plates. In other words, the above process tends to generate dense twin and phase boundaries. Overall, these critical features of TMT enable the systems to deform sequentially by massive amount of SFs, γ → ε, and ε → γ/γ_tw_ phase transformations, enriching the dynamic Hall-Petch effect.

### Material parameters for tailoring TMT in metastable alloys

**Fig. 4.**
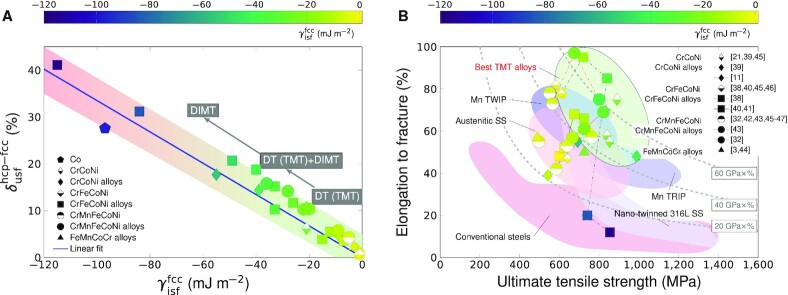
Correlations between the SFE, deformation structure and plastic performance. (A) The calculated $\gamma ^{\rm fcc}_{\rm isf}$ versus $\delta ^{\rm hcp-fcc}_{\rm usf}$ relation for various metastable metals and alloys. The color of the data point shows the size of the corresponding $\gamma ^{\rm fcc}_{\rm isf}$ according to the color code. The shade behind data points is used for guiding eyes. Our TMT mechanism predicts that the primary deformation mechanism changes from DT to DIMT+DT and then to DIMT with decreasing $\gamma ^{\rm fcc}_{\rm isf}$, in perfect agreement with experimental observations (4, 7, 11,17, 18,32, 38–44). (B) Correlation between $\gamma ^{\rm fcc}_{\rm isf}$ and tensile properties. The measured room-temperature elongation to failure (EL,%) is plotted as a function of the ultimate tensile strength (UTS, MPa) for the studied metastable alloys from Refs.(3,11, 21, 32, 38–47), compared to the typical property ranges for conventional steels (48), austenitic stainless steels (SS) (13,49), Mn TRIP and TWIP steels (5,48). Nice correlation can be observed for the excellent mechanical properties and the SFE interval for the effectively activated TMT mechanism in (A). The UTS×EL lines of 20, 40, and 60 GPa×% are shown as thick dashed lines. All data are listed in Table S1. More details about figure preparation are available in Note S4.

In order to establish the general criteria for the activation of TMT, here we analyze the $\gamma ^{\rm fcc}_{\rm isf}$ versus $\delta _{\rm usf}^{\rm hcp-fcc}$ relation for a group of metastable metals and alloys (Fig. 4A and Table S1). It is found that $\delta _{\rm usf}^{\rm hcp-fcc}$ increases linearly with decreasing $\gamma ^{\rm fcc}_{\rm isf}$. Decreasing the negative $\gamma ^{\rm fcc}_{\rm isf}$ provides more thermodynamic driving force for the first step of TMT (γ → SF/ε), but simultaneously making the second step (ε → γ_tw_) more difficult due to the higher $\gamma _{\rm sf}^{\rm hcp}$ and $\gamma _{\rm usf}^{\rm hcp}$, which suppress the nucleation of partials at grain boundaries or the dissociation of full dislocations on the basal planes. Therefore, the TMT mechanism is most efficient for a range of small negative $\gamma ^{\rm fcc}_{\rm isf}$ accompanied by small $\delta _{\rm usf}^{\rm hcp-fcc}$. Accordingly, the primary deformation mode should gradually change from DT(TMT) to DT(TMT)+DIMT and then to DIMT with decreasing $\gamma ^{\rm fcc}_{\rm isf}$, which is in perfect agreement with the experimental observation (see the prevailing deformation modes summarized in Table S1). In particular, when comparing CrCoNi with CrMnFeCoNi, we find that both have small $\delta _{\rm usf}^{\rm hcp-fcc}$ values (6.2% and 4.3%, respectively), the $\gamma ^{\rm fcc}_{\rm isf}$ of CrCoNi (−21 mJ m^−2^) is less than that of CrMnFeCoNi (−5 mJ m^−2^), suggesting that the first step of TMT in CrCoNi is more pronounced than in CrMnFeCoNi (Note S2). Additionally, the larger $\delta _{\rm usf}^{\rm hcp-fcc}$ of CoCrNi than that of CrMnFeCoNi may be responsible for the larger critical twinning stress, which otherwise cannot be expected from the experimental SFEs of the two alloys (22). This observation clearly deciphers the underlying mechanism of the higher twinning propensity and thicker hcp nanolaths in CrCoNi, and thus the superior mechanical performance (7, 22). When the $\gamma ^{\rm fcc}_{\rm isf}$ is further decreased, the deformation-induced ε phase becomes increasingly stable and the second step of TMT will be strongly suppressed. Experimentally these alloys are usually observed as deforming by DIMT, e.g., Co, high-Co HEAs (32, 38) and stainless steels (12, 13), but TMT still occurs in a modest fashion. In the case of metastable fcc Co with very negative SFE ($\gamma ^{\rm fcc}_{\rm isf}=-97$ mJ m^−2^ at 300K), $\delta _{\rm usf}^{\rm hcp-fcc}$ is as large as ∼28%, which impedes the ε → γ_tw_ transformation, and thus DT is least expected in normal tensile experiments (Note S3) (10). However, under severe deformation conditions TMT can still occur in fcc Co grains as evidenced by the characteristic γ/γ_tw_/ε lamellar structure (10).

In order to reach synergy of high strength and high ductility, the present findings suggest that the fcc alloy be tuned metastable by composition design. In other words, the theoretical SFE should be decreased to the negative region to activate TMT twinning. Considering the strong correlation between the stability of the hcp phase and the theoretical SFE, stabilizing the hcp structure is the direct way to decrease the SFE if one cannot access the theoretical SFE value. However, the SFE should not be too negative to avoid the formation of a large fraction of stable ε martensite, which can causes premature fracture (11,14,38). From the correlation between the theoretical SFE and the observed deformation modes summarized in Fig. 4(A), one observes that the deformation microstructure gradually becomes dominant by ε martensite at large negative SFEs. Indeed, for all the metastable alloys investigated here [Fig. 4(B), Table S1], the best mechanical performance is obtained for the alloys within a proper range of negative SFE values, i.e., approximately between −10 and −50 mJ m^−2^. Remarkably, the best “TMT alloys” possess exceptional combination of strength and ductility (>40 to 60 GPa×%) as well as work hardening ability (see Figure S4 for the correlation plot between $\gamma _{\rm isf}^{\rm fcc}$, the difference between ultimate tensile strength and yield strength, and ductility for coarse-grained alloys in Suppplementary Material), surpassing most of the conventional steels and alloys [<20 GPa × % (48)] and even the high performance fully austenitic stainless steel [316L, ∼40 GPa × %(49)].

## Discussion

**Fig. 5.**
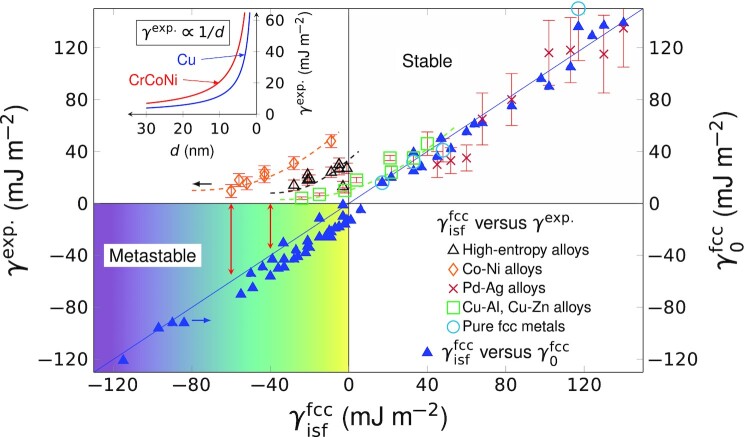
Comparison between theoretical and experimental SFEs ($\gamma _{\rm isf}^{\rm fcc}$ versus *γ*^exp.^). The linear relation between $\gamma ^{\rm fcc}_{\rm isf}$ and $\gamma ^{\rm fcc}_{0}$ for all the studied metals and alloys shows that the two quantities correlate well with each other and that decreasing $\gamma ^{\rm fcc}_{\rm isf}$ indicates less stable fcc phase compared to the hcp phase. The experimental SFE (γ^exp.^) is always positive and fails to reflect the true thermodynamic stability of the fcc structure in metastable alloys. The insert shows the inverse relationship between *γ*^exp.^ and partial separation *d*, which underlies the experimental methods for SFE measurements (53, 52). All explicit values and references are given in Tables S1 and S2, and details about figure preparation are available in Note S4.

**Fig. 6.**
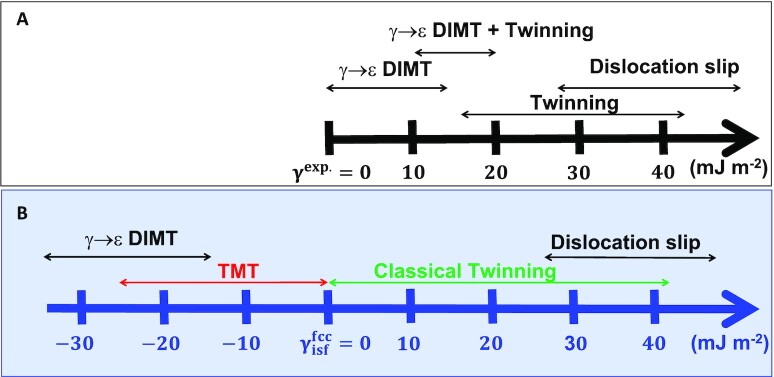
SFE—deformation mode relationship. (A) Empirical relationship between experimental SFE and deformation mode (5). (B) Revised relationship between theoretical SFE and deformation mode.

The present findings shed light on a serious drawback met by the experimental SFE (γ^exp.^) in the realm of metastable alloys (50). Since an intrinsic stacking fault can be viewed as a two-layer embryo of hcp structure embedded in the fcc matrix, the SFE is traditionally connected to the hcp-fcc structural energy difference (Δ*G*^γ → ε^) through $\gamma ^{\rm fcc}_{\rm isf}=2\Delta G^{\gamma \rightarrow \varepsilon }/A+2\sigma$, where *A* is the SF area and σ is the fcc/hcp coherent interfacial energy having the magnitude of a few mJ m^−2^(51). Thus the first order approximation, $\gamma ^{\rm fcc}_{0}\equiv 2\Delta G^{\gamma \rightarrow \varepsilon }/A$, directly reflects the fcc versus hcp structural stability. Indeed, an almost perfect correlation between the theoretical $\gamma _{\rm isf}^{\rm fcc}$ and $\gamma ^{\rm fcc}_{0}$ can be observed in Fig. 5 for various stable and metastable metals and alloys. In contrast, the experimental SFEs (γ^exp.^) only agree with $\gamma _{\rm isf}^{\rm fcc}$ and $\gamma ^{\rm fcc}_{0}$ in stable systems ($\gamma ^{\rm fcc}_{0}\gt 0$), but fail to correlate with the relative thermodynamic stability of the fcc structure in metastable materials ($\gamma ^{\rm fcc}_{0}\lt 0$). The trend that γ^exp.^ approaches zero with decreasing $\gamma ^{\rm fcc}_{0}$ comes from its inverse relationship to the measured partial separation distance *d* (52) or the stacking fault probability (53). According to theory of dislocations (54), γ^exp.^ is assumed to equal to the elastic repulsive force (F_*int*_) between the leading and trailing partial dislocations at equilibrium when the SFE is positive and the lattice friction force is negligible, i.e., γ^exp.^ = *F_int_* = *f*/*d*, where *f* is a positive material parameter depending on the dislocation character (50). However, in metastable alloys the negative excess formation energy of SF can not balance the repulsive force experienced by the partials because they both point to the same direction, and thus the presumed condition (i.e., positive SFE) behind all the existing experimental methods for the evaluation of the SFE breaks down (50, 52, 53). Hence, the most critical factor affecting the nucleation and gliding of partial dislocations, i.e., the true SFE, is not accessible by the current experimental methods in metastable systems. On the other hand, DFT methods predict the SFEs consistently in both stable and metastable alloys. In addition, theory gives access to the intrinsic energy barriers for different deformation modes via the γ-surface, which is also beyond the current experiments. The present findings allow us to reestablish the correlation between the SFE and the prevalent deformation modes (Fig. 6), which paves the road to design plasticity of alloys based quantum mechanical calculations.

## Summary

The disclosed TMT mechanism provides a solid physics-based and universal understanding of the exceptional twinnability and the formation of nanolamella deformation structures in metastable fcc materials. Despite that the reverse ε → γ transformation in hcp metals [e.g., Co (10, 29), Ti (55), and Hf (56)] has long been observed and can be expected from crystallographic relationship between the two crystal structures, the two-step transformation (γ → ε → *γ*_tw_) has never been considered as a critical mechanism underpinning the unusual twinnability in metastable fcc alloys, particularly in TWIP steels, MEAs, and HEAs. The unfortunate situation is partly because of the limitations of experiments in determining the true SFE in metastable materials, i.e., which has badly impeded researchers to capture the thermodynamic stability correctly. Here, using quantum-mechanical DFT calculations, we get access to the critical material parameters that controls the two-step phase transformation processes, i.e., $\gamma ^{\rm fcc}_{\rm isf}$ and $\delta _{\rm usf}^{\rm hcp-fcc}$, with a high resolution enabling the reestablishment of the composition-SFE-deformation mechanism relationship. The present findings advance the current knowledge on the theory of plasticity in metastable fcc materials and guide the design of advanced high strength materials in the infinite composition space to overcome the strength–ductility trade-off.

## Methods

### Calculations

Ab initio calculations based on DFT were performed using the exact muffin-tin orbitals method (57). The exchange-correlation functional was described with the generalized gradient approximation (58). The Kohn–Sham equations were solved within the scalar-relativistic and soft-core schemes. The chemical and magnetic disorders were taken into account using the coherent potential approximation (57). The free energies at room temperature included the lattice expansion and magnetic entropy terms. For metals and alloys with Curie temperature higher than room temperature, ferromagnetic calculations were performed; whereas systems with low Curie temperatures were described in the paramagnetic state modeled by the disordered local magnetic moment approximation (59).

The γ-surfaces in both fcc and hcp lattices were calculated using the hexagonal supercells with 12 close-packed atomic layers along the $\bf c$ (<111 > _fcc_ or <0001 > _hcp_ ) direction. The generalized stacking fault structures were obtained by tilting $\bf c$ axis along the $\lt 11\bar{2}$>_fcc_ or <$\bar{1}010\gt _{\rm hcp}$ directions, respectively, by a shear vector $\bf u$, i.e., $\bf c =\bf c +\bf u$, where $\bf u$ is from 0 to one Burgers vector of the Shockley partial dislocation (b_*p*_). For the calculation of the γ-surface for the cTW route, first we calculated the total energy change of the supercell with atomic stacking of ABCABCABC from $\bf u=0$ to $\bf u=b_p$, from which we obtained $\gamma _{\rm usf}^{\rm fcc}$ and $\gamma _{\rm isf}^{\rm fcc}$. Then for the same supercell we changed the stacking sequence to BCABCABC|B to include one SF, we calculated the total energy change from $\bf c =\bf c +\bf b_p$ to $\bf c =\bf c +2\bf b_p$ and obtained the second half curve of the γ-surface for the cTW route and the $\gamma _{\rm utw}^{\rm fcc}$. Similarly, for getting the γ-surface along the fcc→hcp route, a supercell with one SF, BCABCAB|AB, was adopted at $\bf u=b_p$. Similar methods applied for the calculation of the γ-surfaces in the hcp phase. The numerical parameters were set so that the error bar in the computed intrinsic material parameters is below ∼2 mJ m^−2^.

### Experiments

The Co_32.3_Cr_36.3_Ni_31.4_ (at.%) ingot with 40 mm in diameter was cast by the induction melting technique, homogenized at 1473 K for 8 h and then quenched in water. Plates with thickness of 20 mm were cold rolled to 2 mm. The rolled sheet was annealed at 1273 K for 1 h to obtain a mean grain size of 12 μm. Note that the grain size was measured by using the linear intercept method by counting all the high-angle grain boundaries and twin boundaries. Tensile specimens with gauge length of 10 mm, width of 4 mm, and thickness of 5 mm were cut from the sheets. Tensile tests were conducted at an initial strain rate of 8.3 × 10^−4^ s^−1^ at ambient temperature. The strain was measured by an extensometer until fracture. The microstructures of the Co_32.3_Cr_36.3_Ni_31.4_ alloy after tensile tests were characterized by transmission electron microscope (TEM, FEI Tecnai G2 F20). An accelerating voltage of 200 kV was applied. TEM foils were prepared using a twin-jet electropolishing method by Tenupole-5 in a solution of 90% methanol and 10% perchloric acid at the voltage of 20 V and temperature of 253 K.

## Supplementary Material

pgac282_Supplemental_Files

## Data Availability

All data generated or analyzed during this study are included in this published article (and its supplementary information files).

## Notes

**Competing Financial Interests:** The authors declare no competing financial interests.
