# Supplementary material for: Theory of transformation-mediated twinning
Source: PNAS Nexus. 2022 Dec 7;2(1):pgac282. doi: 10.1093/pnasnexus/pgac282 (PMC9830949; doi:10.1093/pnasnexus/pgac282)
Supplement: pgac282_Supplemental_Files [file pgac282_supplemental_files.zip › PNASNEXUS-PNASNEXUS-2021-00069-s01.pdf]

Supplementary information for

Theory of transformation-mediated twinning

Song Lu∗, Xun Sun∗, Yanzhong Tian, Xianghai An, Wei Li, Yujie Chen, Hualei Zhang∗ and Levente Vitos

To whom correspondence should be addressed: songlu@kth.se (S.L.); xunsun@cityu.edu.hk (X.S.);hualei@xjtu.edu.cn (H.L.Z.)

This PDF file includes:

Supplementary text: Note S1-S4
Fig. S1 to S4
Table S1 and S2
References

Supplementary text

Note S1. The θ values in the matrix and twin parts

For the deformation-induced $\gamma/\gamma_{\mathrm{tw}}$ structure, the θ values on the {111} slip planes parallel to the coherent twin boundary in the matrix (θ^{M}) and twin (θ^{T}) parts are related by $\theta^{\mathrm{T}} = 60^{\circ} - \theta^{\mathrm{M}}$ because the twinning directions in the matrix and twin parts deviate by 60°. Therefore, for CrCoNi when θ^{M} is in the range of 0°-26°, θ^{T} is in the range of 60°-34°. It indicates that the preferred deformation mode is the full dislocation slip in the fresh twin part, but with a significant larger EEB than the stacking fault formation in the matrix, $\overline{\gamma}_{\mathrm{SF}}(\theta^{\mathrm{T}}) > \overline{\gamma}_{\mathrm{SF}}(\theta^{\mathrm{M}})$.

Note S2. Effective energy barriers for CrMnFeCoNi HEA

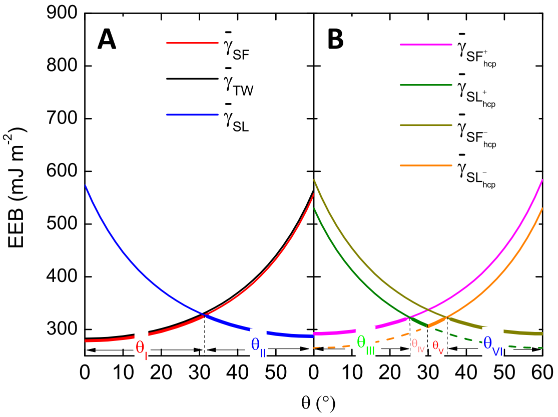

Fig. S1. Calculated EEBs in fcc (A) and hcp (B) CrMnFeCoNi HEA.

In the case of CrMnFeCoNi, both ab initio calculations and experiments show a higer $\gamma_{\mathrm{isf}}^{\mathrm{fcc}}$ than that of CrCoNi [1–7]. The hcp polymorphism was observed at high pressure [8, 9], but at ambient conditions and cryogenic temperatures, only very rare/extremely thin hcp laths were found in normal tensile experiments [2]. Noticeably, the observed thin hcp layers at cryogenic temperature occur also on the coherent twin boundaries [2], an indication for the operation of TMT. Firstly, the small negative $\gamma_{\mathrm{isf}}^{\mathrm{fcc}}$ indicates that the thermodynamic driving force for DIMT is weak and less deformation-induced hcp phase is expected, compared to the case of CrCoNi. Secondly, the particular shape of γ-surface with almost equal $\gamma_{\mathrm{usf}}^{\mathrm{fcc}}$ and $\gamma_{\mathrm{utw}}^{\mathrm{fcc}}$ leads to similar probability for the DIMT and for the conventional layer-by-layer twinning. Thirdly, the orientation window for the retained hcp ($\theta_I - \theta_{III}$) in Fig.S1 is limited to 26°-31°, i.e., ∼38% smaller than that in CrCoNi. Overall, it leads to the conclusion that the deformation-induced hcp phase, if it indeed occurs in the severe competition with normal twinning, are potentially subjected to further $\varepsilon \rightarrow \gamma_{\mathrm{tw}}$ transformation, and then barely any hcp phase survives in actual observation.

The obtained γ-surfaces for $Cr_{16}Co_{40}Ni_{44}$, $Cr_{25}Fe_{30}Co_{20}Ni_{25}$, $Cr_{25}Fe_{20}Co_{30}Ni_{25}$, $Cr_{25}Fe_{15}Co_{35}Ni_{25}$, CrFeCoNi, CrMnFeCoNi and $Cr_{10}Mn_{40}Fe_{40}Co_{10}$ are very similar as listed in Table S1, as well as the observed primary deformation mechanism at room temperature, dominantly by DT [10–13]. Decreasing temperature can decrease the γ_{isf} of these alloys, which will promote TMT. For example, at cryogenic deformation temperatures such as 77K and 4.2K, in CoFeCoNi the $\gamma/\gamma_{\mathrm{tw}}/\varepsilon$ lamellar structure was clearly resolved by high-angle annular dark-field scanning TEM, indicating the operation of TMT mechanism [12]. Additionally, one may decrease the $\gamma_{\mathrm{isf}}^{\mathrm{fcc}}$ by tuning composition to further promote TMT. For instance, increasing Co content (and decreasing Ni) in the Cr-Mn-Fe-Co-Ni alloys, the primary deformation modes were shown to change from DT to DT+DIMT, accompanied with simultaneously improved strength and ductility [14].

Note S3. Effective energy barriers for Co

The calculated EEBs in fcc and hcp Co are plotted in Fig.S2. Because of the big negative $\gamma_{\mathrm{isf}}^{\mathrm{fcc}}$, stacking faults and fcc→ hcp phase transformation can easily occur during deformation, which is also reflected by the broad range of $\theta_I = 0^{\circ} - 45^{\circ}$ in Fig.S2A. The $\delta_{\mathrm{usf}}^{\mathrm{hcp-fcc}}$ is however, very high, ∼28%, indicating that subsequent $\varepsilon \rightarrow \gamma_{\mathrm{tw}}$ transformation can not easily happen. Nevertheless, under extreme conditions such as high-pressure torsion (HPT) [15] and surface mechanical attrition treatment [16, 17], the TMT chain deformation process $\gamma \rightarrow \varepsilon \rightarrow \gamma_{\mathrm{tw}}$ still occurs, as evidenced by the observed $\gamma/\gamma_{\mathrm{tw}}/\varepsilon$ lamellar structure in the fcc grains [16, 17]. The same experiments have also shown extensive stacking faults on the basal planes in the hcp grains [16, 17]. Nano-sized fcc phase in the hcp Co grains was also observed in molecular dynamic simulations [18]. Edalati et al. [15] showed that during HPT processing of Co, the fcc→ hcp transformation occurs until the average grain size reaches submicrometer level, but the reverse hcp→ fcc phase transformation occurs when the grain size is reduced to the nanometer level. Temperature rise during HPT was ruled out as the reason [15]. The above experimental results are consistent with our prediction in Fig.S2b, showing basal stacking

fault formation for θ in the ranges of 0°-18° and 42°-60°. Notice that in the hcp grains of Co, the $\{10\bar{1}1\}$ deformation twins and <c+a> dislocations compete strongly with dislocation activities on the basal planes, which may also suppress TMT [16–18].

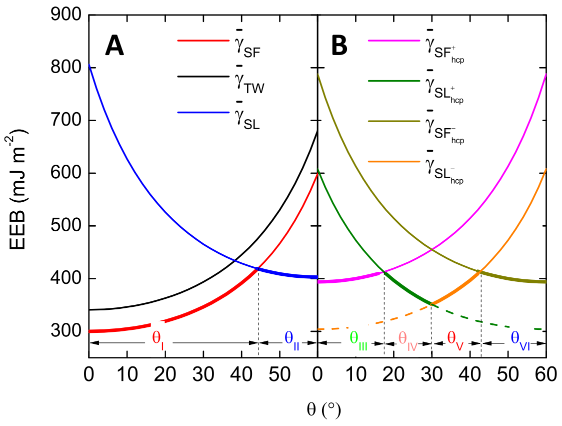

Fig. S2. Calculated EEBs in fcc (A) and hcp (B) Co.

Note S4. Details about figure preparation

Fig.1A and 1B show the γ-surfaces in fcc and hcp. The computational method for γ-surfaces are described in the method section. The critical values of stacking fault energy, and unstable stacking fault energies for CrCoNi are listed in Table S1. The curves are cosine interpolations between calculated values.

Fig.1C shows the EEBs for stacking fault formation (SF), full dislocation slip (SL) and classical twinning (cTW) with respect to θ (0-60°) which are calculated according to Eq. (1)-(3), respectively. For $0^o \leq \theta < 34^o$, the EEB for stacking fault is the lowest one, then we denote this range as θ_I and color it with light red. For $34^o \leq \theta < 60^o$, the EEB for SL is the lowest one, then we denote this range as θ_{II} and color it with light blue.

Fig.1D shows the EEBs for deformation modes in hcp structure which are calculated according to Eq. (4-7) and plotted in the similar way as for fcc.

Fig.1E schematically shows the atomic positions in the three consecutive fcc (111) layers, i.e., ABC. The structure is viewed from [111] direction with C being the top layer. We differ the distances of the three layers from our eyes by using different sizes of circles surrounding the ABC letters, with the C layer being the closest, therefore the largest circle. On the figure, we mark the three Burgers vectors for leading partials with red arrows and the other three Burgers vectors for trailing partials with blue dashed arrows. Since θ is defined from a twinning direction, here we choose the [11-2] direction (i.e., b_1^{fcc} on (111) planes as the reference direction. So θ tells the direction of the resolved shear stress relative to b_1^{fcc} and spans 0°-60°, i.e., the range of angle between b_1^{fcc} and b_6^{fcc}. We color correspondingly the θ_I range with the same light red color in as Fig.1c. The θ_{II} range which favors the activation of full dislocation slip is indicated by blue color in Fig.1c, and 1e.

Fig.1F schematically shows the atomic positions in the two consecutive hcp (0001) layers, i.e., AC, similarly as for fcc in Fig.1e. The three Burgers vectors for leading partials slipping on basal plane C are denoted as b_1^{hcp}, b_2^{hcp} and b_3^{hcp} by red arrows. They are equivalent to those for leading partials in fcc (Fig.1e). The other three Burgers vectors for leading partials slipping on basal plane A are denoted as b_4^{hcp}, b_5^{hcp} and b_6^{hcp}. b_1^{hcp} and b_1^{fcc} are identical and parallel to each other because of the Shoji-Nishiyama (S-N) orientation relationship between the fcc matrix and the deformation-induced ε martensite. So here θ measures the direction between the resolved shear stress on the basal planes relative to b_1^{hcp} and also spans 0°-60°.

By plotting θ_I to θ_{VI} ranges obtained in Fig.1c and 1d in Fig.1e and 1f, one easily sees that which dislocation slip (characterized by the corresponding Burgers vector) is preferred and the shift of atoms.

Fig.4A plots the relationship between $\delta_{\rm usf}^{\rm (hcp-fcc)} = (\gamma_{\rm usf}^{\rm hcp} - \gamma_{\rm usf}^{\rm fcc})/\gamma_{\rm usf}^{\rm fcc}$ and $\gamma_{\rm isf}^{\rm fcc}$ for the various alloys. All the values are listed in Table S1. To guide eyes, we use a color code to show the size of $\gamma_{\rm isf}^{\rm fcc}$, and each data point is colored accordingly. The background shade behind the data points is used to emphasize the linear relationship between $\delta_{\rm usf}^{\rm (hcp-fcc)}$ and $\gamma_{\rm isf}^{\rm fcc}$ (the background color has no further meaning).

Fig.4B plots the total elongation and ultimate tensile strength data taken from references for the studied alloys (all data is available in the Table S1). The SFE values of the alloys are shown by the color of the data points according to the color code, which is the same as in Fig.4a. The background is the overview diagram showing some typical value ranges for the total elongation to fracture and the ultimate tensile tensile strength for a number of different classes of steels. The readers are referred to Figure 8 in Ref. [19] by Raabe et al. and the relevant references there for more details about the overview diagram.

Fig.5 plots the relationships between the calculated $\gamma_{\rm isf}^{\rm fcc}$, $\gamma_0^{\rm fcc}$ and the experimental stacking fault values for various alloys. All the values are listed in the Tables S1 and S2. The dashed lines behind the experimental stacking fault energy data points are for guiding eyes. At the left corner of Fig.5 where $\gamma_{\rm isf}^{\rm fcc}$ is negative, we use gradient color background to reflect the negative stacking fault energy, which is similar to the color bar for SFE in Fig.4. The insert plot shows how the experimental SFE changes with the

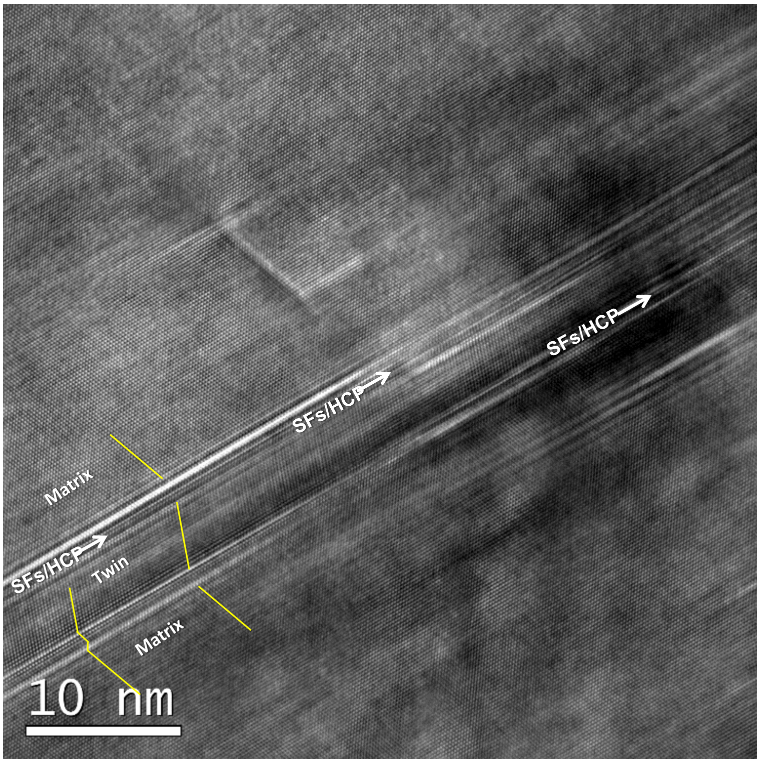

Fig. S3. TEM images of microstructures after room temperature tensile testing till necking in a $Cr_{32.3}Co_{36.3}Ni_{31.4}$ MEA, showing the typical lamellar structure composed of fcc matrix, twin, and hcp nanolayers.

partial separation width d to reflect the $\gamma^{\mathrm{exp.}} \propto G/d$ relationship. Here G is the shear modulus. The two curves for CrCoNi and Cu are different due to the fact that the shear modulus of CrCoNi is higher than Cu. Readers are referred to Ref.[20] for detailed discussions about the experimental SFE measurements.

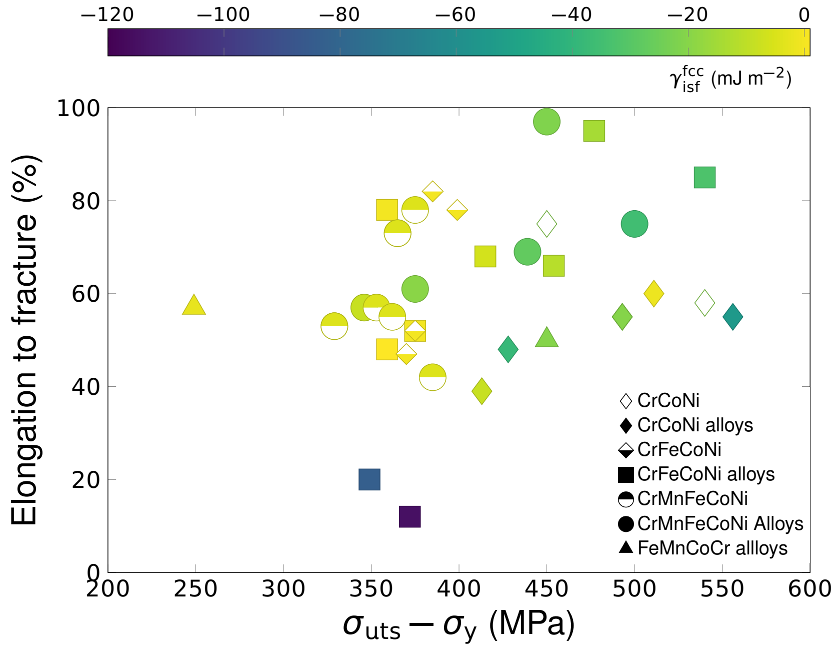

Fig. S4. Correlation between γ_{isf}^{fcc} and tensile properties. The measured room-temperature elongation to failure (EL,%) is plotted with respect to the difference between the ultimate tensile strength (UTS, MPa) and the yield strength ($\sigma_{0.2}$, MPa) for coarse-grained alloys available in literature. The color of the data point shows the corresponding SFE. All the data and the references are listed in Table S1.

Table S1. Theoretical generalized stacking fault energies (GSFE, mJ m^{-2}) for various metals and alloys in both fcc and hcp structures, in comparison with available theoretical (Calc.) and experimental (Exp.) data in the literature. The room temperature GSFEs are calculated at the corresponding experimental lattice parameter of the fcc structure or at the lattice parameter estimated by a regression formula [21] in case that the experimental value is missing. Paramagnetic (PM) calculations are performed, except for Co and Co-rich alloys which are ferromagnetic (FM) at 300K. The nonmagnetic (NM) results in the literature are also included for comparison. Values of $\gamma_0^{\rm fcc}$ and $\delta_{\rm usf}^{\rm hcp-fcc}$ are listed (see main text for definition). The reported primary deformation mechanisms (DT and/or DIMT) during normal tensile tests at both room and cryogenic temperatures are indicated. The measured elongation to failure (EL,%), ultimate tensile stress (UTS, MPa) and yield strength ($\sigma_{0.2}$) for coarse-grained alloys at room temperature in the literature are listed. All concentrations are given in at.%.

		Magnetic			fcc				hcp			Deformation			
	T	state	a (Å)	$\gamma_{\rm usf}^{\rm fcc}$	$\gamma_{\rm isf}^{\rm fcc}$	$\gamma_{\rm utf}^{\rm fcc}$	$\gamma_{\rm esf}^{\rm fcc}$	$\gamma_{\rm usf}^{\rm hcp}$	$\gamma_{\rm isf}^{\rm hcp}$	$\gamma_0^{\rm fcc}$	$\delta_{\rm usf}^{\rm hcp-fcc}$	mode	UTS	$\sigma_{0.2}$	EL (%)
Co	300K	FM	3.538[22]	297	-97	234	-116	379	86	-96	27.6%				
Co	0K	FM	3.529	300	-103	238	-119	388	87	-100	29.3%				
Calc.[2]	0K	FM	3.522		-137		-111			-89					
Calc.[2]	300K	FM								-67					
Exp.									27±4[23]			DIMT			
									31±5[24]						
CrCoNi	300K	PM	3.567[5]	306	-21	295	-13	325	35	-29	6.2%				
CrCoNi	0K	PM	3.526	350	-29	336	-18	382	56	-43	9.1%				
Calc.[25]	0K	NM	3.526		-43		-28								
Calc.[26]	0K	NM	3.516	264	-24	310	-17		-						
Calc.[4]	0K	FM	3.532	280	-62		-42			-40					
Calc.[2]	0K	FM		287	-38	251	-20			-58					
Calc.[2]	300K	FM								-53					
Exp.			3.529[7]		18±4[7]							DIMT+DT[2, 27, 28]			
			3.567[5]		22±4[5]										
													840	300	58[29]
													853	360	55[30]
													890	440	75[31]
$Cr_{16}Co_{40}Ni_{44}$	300K	FM	3.551	335	-9	335	5	351	41	-26	4.8%	DT[10]	542	129	39[10]
$Cr_{16}Co_{54}Ni_{30}$	300K	FM	3.548	334	-55	309	-35	393	87	-70	17.7%	DIMT[10]	692	136	55[10]
$Cr_{25}Co_{37.5}Ni_{37.5}$	300K	PM	3.561	323	-2	324	7	329	28	-16	1.9%		750	247	60[30]
$Cr_{45}Co_{27.5}Ni_{27.5}$	300K	PM	3.584	271	-39	249	-35	310	48	-43	14.4%		998	560	48[30]
CrFeCoNi	300K	PM	3.577[21]	299	-1	298	7	301	26	-13	0.7%				
Calc.[4]	0K	NM	3.540	240	-23					-25					
Exp.			3.565[7]		27±4[7]							DT[11, 12]			
												DIMT+DT (77K)[12]			
													625	250	52[11]
													640	270	47[29]
													582	197	82[32]
													786		49[32]
													610	211	78[33]
$Cr_{25}Fe_{30}Co_{20}Ni_{25}$	300K	PM	3.580	290	1	291	10	294	25	-13	1.4%	DT[11]	600	241	48[11]
$Cr_{25}Fe_{20}Co_{30}Ni_{25}$	300K	PM	3.573	304	-7	302	1	315	29	-19	3.6%	DT[11]	675	260	68[11]
$Cr_{25}Fe_{15}Co_{35}Ni_{25}$	300K	PM	3.569	310	-12	305	-3	327	38	-26	5.5%	DT[11]	725	271	66[11]
$Cr_{25}Fe_{25}Co_{35}Ni_{15}$	300K	PM	3.575	291	-26	279	-15	325	55	-41	11.7%	DIMT[34]	750		65[34]
$Cr_{25}Fe_{30}Co_{35}Ni_{10}$	300K	PM	3.578	290	-33	275	-21	334	64	-49	15.2%	DIMT[34]	825		50[34]
$Cr_{25}Fe_{35}Co_{35}Ni_{5}$	300K	PM	3.580	292	-40	274	-28	347	73	-56	18.8%	DIMT[34]	900		42[34]
$Cr_{25}Fe_{40}Co_{35}Ni_{0}$	300K	PM	3.583	294	-49	271	-35	355	81	-65	20.7%	DIMT[34]	1000		35[34]
$Cr_{25}Fe_{20}Co_{35}Ni_{20}$	300K	PM	3.572	306	-15	298	-6	318	34	-26	3.9%	DT[33]	720	243	95[33]
$Cr_{25}Fe_{15}Co_{45}Ni_{15}$	300K	PM	3.568	312	-33	296	-23	344	48	-43	10.3%	DIMT[33]	840	300	85[33]
$Cr_{25}Fe_{10}Co_{55}Ni_{10}$	300K	FM	3.563	291	-84	246	-82	382	98	-92	31.3%	DIMT[33]	741	392	20[33]
$Cr_{25}Fe_{5}Co_{65}Ni_{5}$	300K	FM	3.559	292	-115	230	-114	412	124	-121	41.1%	DIMT[33]	854	482	12[33]
CrMnFeCoNi	300K	PM	3.594[35]	280	-5	278	4	292	30	-18	4.3%				
Calc.[2]	0K	FM		374	-31	313	3			-43					
Calc.[2]	300K	FM								-26					
Calc.[4]	0K	FM	3.541		-54					-25					
Exp.			3.576[7]		26.5±4.5[7]							DT[36]			
			3.606[6]		30±5[6]							DT+DIMT (77K)[2]			
													550	175	78[37]
													763	410	57[36]
													497	135	55[32]
													851		25[32]
													585	200	42[29]
													625		60[37]
													565	200	73[38]
													639	310	53[14]
$Cr_{20}Mn_{15}Fe_{15}Co_{35}Ni_{15}$	300K	PM	3.582	290	-22	279	-12	320	52	-38	10.3%	DT[38]	675	225	97[38]
$Cr_{25}Mn_{15}Fe_{10}Co_{35}Ni_{15}$	300K	PM	3.585	285	-36	268	-24	330	64	-50	15.8%	DIMT[38]	800	300	75[38]
$Cr_{20}Mn_{20}Fe_{20}Co_{23}Ni_{17}$	300K	PM	3.598[14]	276	-9	272	0	292	35	-22	5.8%	DT[14]	666	320	57[14]
Exp.					24±4[14]										
$Cr_{20}Mn_{20}Fe_{20}Co_{27}Ni_{13}$	300K	PM	3.595[14]	279	-20	270	-9	308	48	-34	10.4%	DT[14]	725	350	61[14]
Exp.					19±3[14]										
$Cr_{20}Mn_{20}Fe_{20}Co_{30}Ni_{10}$	300K	PM	3.593[14]	282	-28	269	-17	322	58	-43	14.2%	DT+DIMT[14]	821	382	69[14]
Exp.					14±4[14]										
$Cr_{10}Mn_{30}Fe_{50}Co_{10}$	300K	PM	3.600[39]	278	-21	267	-8	308	54	-33	10.8%	DIMT+DT [39]			
													880		75[40]
													725	275	50[40]
Calc.[39]	300K	NM								-23					
$Cr_{10}Mn_{40}Fe_{40}Co_{10}$	300K	PM	3.621[13]	260	-3	257	4	266	23	-10	2.3%	DT[13]			
Exp.			3.610[41]		13±4[41]							DT+DIMT[42]	489	240	57[13]

Table S2. Theoretical and experimental SFEs for pure fcc metals and alloys. The calculated γ_{isf}^{fcc} and γ_0^{fcc} for fcc metals, Cu-Al, Cu-Zn and Pd-Ag alloys are from Refs. [43–45].

Systems (at.%)	γ_{isf}^{fcc} (mJ m^{-2})	γ_0^{fcc} (mJ m^{-2})	$\gamma_{exp.}$ (mJ m^{-2})
Ag	17	16	16±2[46]
Cu	48	49	41±9[46]
Au	33	25	32±5[47]
Al	117	136	150±40[48]
Ni	154	157	125±5[49]
Cu-2.2Al	33	39	35±5[50]
Cu-4.9Al	22	20	24±5[50]
Cu-10Al	-2	-1	10±2[51]
Cu-13.4Al	-15	-11.5	7±1[52]
Cu-20Al	-33	-30	
Cu-2.0Zn	40		46±9[50]
Cu-10Zn	21		35±2[53]
Cu-20Zn	4		18±2[53]
Co-90Ni	124	129	
Co-80Ni	98	96	
Co-70Ni	64	61	
Co-60Ni	37	28	
Co-50Ni	6	-5	
Co-45Ni	-10	-21	48±5[24]
Co-40Ni	-27	-36	31±5[24]
Co-35Ni	-44	-49	24±5[24]
Co-36Ni			21±5[24]
Co-33Ni	-50	-54	16±5[24]
Co-32Ni			18±5[24]
Co-31Ni	-57	-61	10±5[24]
Co-15Ni	-90	-92	
Pd-20Ag	140	139	135±30[54]
Pd-25Ag	130	137	115±30[54]
Pd-35Ag	113	105	118±25[54]
Pd-40Ag	102	90	116±25[54]
Pd-50Ag	83	75	80±20[54]
Pd-60Ag	68	62	65±20[54]
Pd-70Ag	60	55	35±10[54]
Pd-80Ag	52	42	33±10[54]
Pd-90Ag	45	36	30±10[54]

References

1. Duancheng Ma, Blazej Grabowski, Fritz Körmann, Jörg Neugebauer, and Dierk Raabe. Ab initio thermodynamics of the CoCrFeMnNi high entropy alloy: Importance of entropy contributions beyond the configurational one. *Acta Mater.*, 100:90 – 97, 2015.
2. Changning Niu, Carlyn R. LaRosa, Jiashi Miao, Michael J. Mills, and Maryam Ghazisaeidi. Magnetically-driven phase transformation strengthening in high entropy alloys. *Nat. Comm.*, 9(1):1363, 2018.
3. Shuo Huang, He Huang, Wei Li, Dongyoo Kim, Song Lu, Xiaoqing Li, Erik Holmström, Se Kyun Kwon, and Levente Vitos. Twinning in metastable high-entropy alloys. *Nat. Comm.*, 9:2381, 2018.
4. Shijun Zhao, G. Malcolm Stocks, and Yanwen Zhang. Stacking fault energies of face-centered cubic concentrated solid solution alloys. *Acta Mater.*, 134:334 – 345, 2017.
5. G. Laplanche, A. Kostka, C. Reinhart, J. Hunfeld, G. Eggeler, and E.P. George. Reasons for the superior mechanical properties of medium-entropy CrCoNi compared to high-entropy CrMnFeCoNi. *Acta Mater.*, 128:292 – 303, 2017.
6. Norihiko L. Okamoto, Shu Fujimoto, Yuki Kambara, Marino Kawamura, Zhenghao M.T. Chen, Hirotaka Matsunoshita, Katsushi Tanaka, Haruyuki Inui, and Easo P. George. Size effect, critical resolved shear stress, stacking fault energy, and solid solution strengthening in the CrMnFeCoNi high-entropy alloy. *Sci. Rep.*, 6:35863, 2016.
7. S.F. Liu, Y. Wu, H.T. Wang, J.Y. He, J.B. Liu, C.X. Chen, X.J. Liu, H. Wang, and Z.P. Lu. Stacking fault energy of face-centered-cubic high entropy alloys. *Intermetallics*, 93:269 – 273, 2018.
8. Fei Zhang, Yuan Wu, Hongbo Lou, Zhidan Zeng, Vitali B. Prakapenka, Eran Greenberg, Yang Ren, Jinyuan Yan, John S. Okasinski, Xiongjun Liu, Yong Liu, Qiaoshi Zeng, and Zhaoping Lu. Polymorphism in a high-entropy alloy. *Nat. Comm.*, 8:15687, 2017.
9. Cameron L. Tracy, Sulgiye Park, Dylan R. Rittman, Steven J. Zinkle, Hongbin Bei, Maik Lang, Rodney C. Ewing, and Wendy L. Mao. High pressure synthesis of a hexagonal close-packed phase of the high-entropy alloy CrMnFeCoNi. *Nat. Comm.*, 8:15634, 2017.
10. L. Rémy and A. Pineau. Twinning and strain-induced f.c.c. $\rightarrow$ h.c.p. transformation on the mechanical properties of Co-Ni-Cr-Mo alloys. *Mater. Sci. Eng.*, 26(1):123 – 132, 1976.
11. Wei Fang, Ruobin Chang, Xin Zhang, Puguang Ji, Xinghua Wang, Baoxi Liu, Jia Li, Xinbo He, Xuanhui Qu, and Fuxing Yin. Effects of cobalt on the structure and mechanical behavior of non-equal molar $Co_xFe_{50-x}Cr_{25}Ni_{25}$ high entropy alloys. *Mater. Sci. Eng.: A*, 723:221 – 228, 2018.
12. Qingyun Lin, Junpeng Liu, Xianghai An, Hao Wang, Yong Zhang, and Xiaozhou Liao. Cryogenic-deformation-induced phase transformation in an FeCoCrNi high-entropy alloy. *Mater. Res. Lett.*, 6(4):236–243, 2018.
13. Y. Deng, C.C. Tasan, K.G. Pradeep, H. Springer, A. Kostka, and D. Raabe. Design of a twinning-induced plasticity high entropy alloy. *Acta Mater.*, 94:124 – 133, 2015.
14. S.F. Liu, Y. Wu, H.T. Wang, W.T. Lin, Y.Y. Shang, J.B. Liu, K. An, X.J. Liu, H. Wang, and Z.P. Lu. Transformation-reinforced high-entropy alloys with superior mechanical properties via tailoring stacking fault energy. *J. Alloys Compd.*, 792:444 – 455, 2019.
15. Kaveh Edalati, Shoichi Toh, Makoto Arita, Masashi Watanabe, and Zenji Horita. High-pressure torsion of pure cobalt: hcp-fcc phase transformations and twinning during severe plastic deformation. *Appl. Phys. Lett.*, 102(18):181902, 2013.
16. X. Wu, N. Tao, Y. Hong, J. Lu, and K. Lu. $\gamma \rightarrow \varepsilon$ martensite transformation and twinning deformation in fcc cobalt during surface mechanical attrition treatment. *Scr. Mater.*, 52(7):547 – 551, 2005.
17. X. Wu, N. Tao, Y. Hong, G. Liu, B. Xu, J. Lu, and K. Lu. Strain-induced grain refinement of cobalt during surface mechanical attrition treatment. *Acta Mater.*, 53(3):681 – 691, 2005.
18. Wen Wang, Fuping Yuan, Ping Jiang, and Xiaolei Wu. Size effects of lamellar twins on the strength and deformation mechanisms of nanocrystalline hcp cobalt. *Sci. Rep.*, 7:9550, 2017.
19. Dierk Raabe, Cemal Cem Tasan, Hauke Springer, and Michael Bausch. From high-entropy alloys to high-entropy steels. *steel research int.*, 86:1127, 2015.
20. Xun Sun, Song Lu, Ruiwen Xie, Xianghai An, Wei Li, Tianlong Zhang, Chuanxin Liang, Xiangdong Ding, Yunzhi Wang, Hualei Zhang, and Levente Vitos. Can experiment determine the stacking fault energy of metastable alloys? *Mater. & Des.*, 199:109396, 2021.
21. Zhijun Wang, Qingfeng Wu, Wenquan Zhou, Feng He, Chunyan Yu, Deye Lin, Jincheng Wang, and C.T. Liu. Quantitative determination of the lattice constant in high entropy alloys. *Scr. Mater.*, 162:468 – 471, 2019.
22. W. Betteridge. The properties of metallic cobalt. *Prog. Mater. Sci.*, 24:51 – 142, 1980.
23. A. Korner and H. P. Karnthaler. Weak-beam study of glide dislocations in h.c.p. cobalt. *Phil. Mag. A*, 48(3):469–477, 1983.
24. T Ericsson. The temperature and concentration dependence of the stacking fault energy in the Co-Ni system. *Acta Metall.*, 14(7):853 – 865, 1966.
25. Jun Ding, Qin Yu, Mark Asta, and Robert O. Ritchie. Tunable stacking fault energies by tailoring local chemical order in CrCoNi medium-entropy alloys. *Proc. Natl. Acad. Sci.*, 115(36):8919–8924, 2018.
26. Zijiao Zhang, Hongwei Sheng, Zhangjie Wang, Bernd Gludovatz, Ze Zhang, Easo P. George, Qian Yu, Scott X. Mao, and Robert O. Ritchie. Dislocation mechanisms and 3D twin architectures generate exceptional strength-ductility-toughness combination in CrCoNi medium-entropy alloy. *Nat. Comm.*, 8:14390, 2017.
27. C.E. Slone, S. Chakraborty, J. Miao, E.P. George, M.J. Mills, and S.R. Niezgoda. Influence of deformation induced nanoscale twinning and FCC-HCP transformation on hardening and texture development in medium-entropy CrCoNi alloy. *Acta Mater.*, 158:38 – 52, 2018.

28. J. Miao, C.E. Slone, T.M. Smith, C. Niu, H. Bei, M. Ghazisaeidi, G.M. Pharr, and M.J. Mills. The evolution of the deformation substructure in a Ni-Co-Cr equiatomic solid solution alloy. *Acta Mater.*, 132:35 – 48, 2017.
29. Zhenggang Wu, Yanfei Gao, and Hongbin Bei. Thermal activation mechanisms and labusch-type strengthening analysis for a family of high-entropy and equiatomic solid-solution alloys. *Acta Mater.*, 120:108 – 119, 2016.
30. Francisco G. Coury, Kester D. Clarke, Claudio S. Kiminami, Michael J. Kaufman, and Amy J. Clarke. High throughput discovery and design of strong multicomponent metallic solid solutions. *Sci. Rep.*, 8:8600, 2018.
31. Bernd Gludovatz, Anton Hohenwarter, Keli V.S. Thurston, Hongbin Bei, Zhenggang Wu, Easo P. George, and Robert O. Ritchie. Exceptional damage-tolerance of a medium-entropy alloy CrCoNi at cryogenic temperatures. *Nat. Comm.*, 7:10602, 2016.
32. A.J. Zaddach, R.O. Scattergood, and C.C. Koch. Tensile properties of low-stacking fault energy high-entropy alloys. *Mater. Sci. Eng.: A*, 636:373 – 378, 2015.
33. Daixiu Wei, Xiaoqing Li, Weicheng Heng, Yuichiro Koizumi, Feng He, Won-Mi Choi, Byeong-Joo Lee, Hyoung Seop Kim, Hidemi Kato, and Akihiko Chiba. Novel Co-rich high entropy alloys with superior tensile properties. *Mater. Res. Lett.*, 7(2):82–88, 2019.
34. Wei Fang, Ruobin Chang, Puguang Ji, Xin Zhang, Baoxi Liu, Xuanhui Qu, and Fuxing Yin. Transformation induced plasticity effects of a non-equal molar Co-Cr-Fe-Ni high entropy alloy system. *Metals*, 8:369, 2018.
35. J.Y. He, W.H. Liu, H. Wang, Y. Wu, X.J. Liu, T.G. Nieh, and Z.P. Lu. Effects of Al addition on structural evolution and tensile properties of the FeCoNiCrMn high-entropy alloy system. *Acta Mater.*, 62:105 – 113, 2014.
36. Bernd Gludovatz, Anton Hohenwarter, Dhiraj Catoor, Edwin H. Chang, Easo P. George, and Robert O. Ritchie. A fracture-resistant high-entropy alloy for cryogenic applications. *Science*, 345(6201):1153–1158, 2014.
37. F. Otto, A. Dlouhý, Ch. Somsen, H. Bei, G. Eggeler, and E.P. George. The influences of temperature and microstructure on the tensile properties of a CoCrFeMnNi high-entropy alloy. *Acta Mater.*, 61(15):5743 – 5755, 2013.
38. Daixiu Wei, Xiaoqing Li, Jing Jiang, Weicheng Heng, Yuichiro Koizumi, Won-Mi Choi, Byeong-Joo Lee, Hyoung Seop Kim, Hidemi Kato, and Akihiko Chiba. Novel Co-rich high performance twinning-induced plasticity (TWIP) and transformation-induced plasticity (TRIP) high-entropy alloys. *Scr. Mater.*, 165:39 – 43, 2019.
39. Wenjun Lu, Christian H. Liebscher, Gerhard Dehm, Dierk Raabe, and Zhiming Li. Bidirectional transformation enables hierarchical nanolaminate dual-phase high-entropy alloys. *Adv. Mater.*, 30(44):1804727, 2018.
40. Zhiming Li, Konda Gokuldoss Pradeep, Yun Deng, Dierk Raabe, and Cemal Cem Tasan. Metastable high-entropy dual-phase alloys overcome the strength-ductility trade-off. *Nature*, 534(7606):227–230, 2016.
41. S. Picak, J. Liu, C. Hayrettin, W. Nasim, D. Canadinc, K. Xie, Y.I. Chumlyakov, I.V. Kireeva, and Ibrahim Karaman. Anomalous work hardening behavior of Fe40Mn40Cr10Co10 high entropy alloy single crystals deformed by twinning and slip. *Acta Mater.*, 181:555 – 569, 2019.
42. A. K. Chandan, M. Tripathy, S.and Ghosh, and S. G. Chowdhury. Evolution of substructure of a non-equiatomic FeMnCrCo high entropy alloy deformed at ambient temperature. *Metall. Mater. Trans. A*, 50:5079–5090, 2019.
43. Ruihuan Li, Song Lu, Dongyoo Kim, Stephan Schönecker, Jijun Zhao, Se Kyun Kwon, and Levente Vitos. Stacking fault energy of face-centered cubic metals: thermodynamic and ab initio approaches. *J. Phys.: Condensed Matter*, 28(39):395001, 2016.
44. Wei Li, Song Lu, Qing-Miao Hu, Se Kyun Kwon, Börje Johansson, and Levente Vitos. Generalized stacking fault energies of alloys. *J.Phys.: Condensed Matter*, 26(26):265005, 2014.
45. Song Lu, Qing-Miao Hu, Erna Krisztina Delczeg-Czirjak, Börje Johansson, and Levente Vitos. Determining the minimum grain size in severe plastic deformation process via first-principles calculations. *Acta Mater.*, 60(11):4506 – 4513, 2012.
46. D. J. H. Cockayne, M. L. Jenkins, and I. L. F. Ray. The measurement of stacking-fault energies of pure face-centred cubic metals. *Phil. Mag.*, 24(192):1383–1392, 1971.
47. M. L. Jenkins. Measurement of the stacking-fault energy of gold using the weak-beam technique of electron microscopy. *Phil. Mag.*, 26(3):747–751, 1972.
48. Michael J. Mills and Pierre Stadelmann. A study of the structure of lomer and 60° dislocations in aluminium using high-resolution transmission electron microscopy. *Phil. Mag. A*, 60(3):355–384, 1989.
49. C. B. Carter and S. M. Holmes. The stacking-fault energy of nickel. *Phil. Mag.*, 35(5):1161–1172, 1977.
50. C. B. Carter and I. L. F. Ray. On the stacking-fault energies of copper alloys. *Phil. Mag.*, 35(1):189–200, 1977.
51. D. J. H. Cockayne, I. L. F. Ray, and M. J. Whelan. Investigations of dislocation strain fields using weak beams. *Phil. Mag.*, 20(168):1265–1270, 1969.
52. H. Saka, Y. Sueki, and T. Imura. On the intrinsic temperature dependence of the stacking-fault energy in copper-aluminium alloys. *Phil. Mag.A*, 37(2):273–289, 1978.
53. P. C. J. Gallagher. The influence of alloying, temperature, and related effects on the stacking fault energy. *Metall. Trans.*, 1(9):2429–2461, 1970.
54. I. R. Harris, I. L. Dillamore, R. E. Smallman, and B. E. P. Beeston. The influence of d-band structure on stacking-fault energy. *Phil. Mag.*, 14(128):325–333, 1966.
